# TET1 regulates responses to house dust mite by altering chromatin accessibility, DNA methylation, and gene expression in airway epithelial cells

**DOI:** 10.21203/rs.3.rs-3726852/v1

**Published:** 2023-12-13

**Authors:** Anthony P. Brown, Sreeja Parameswaran, Lucy Cai, Sweeney Elston, Chi Pham, Artem Barski, Matthew T. Weirauch, Hong Ji

**Affiliations:** University of California Davis; Cincinnati Children’s Hospital Medical Center; University of California Davis; University of California Davis; University of California Davis; Cincinnati Children’s Hospital Medical Center; Cincinnati Children’s Hospital Medical Center; University of California Davis

**Keywords:** TET1, asthma, transcription factor, chromatin accessibility, methylation, RNA-seq, ATAC-seq, whole genome bisulfite sequencing, functional genomics, gene regulation

## Abstract

**Background:**

Previous studies have identified TET1 as a potential key regulator of genes linked to asthma. TET1 has been shown to transcriptionally respond to house dust mite extract, an allergen known to directly cause allergic asthma development, and regulate the expression of genes involved in asthma. How TET1 regulates expression of these genes, however, is unknown. TET1 is a DNA demethylase; therefore, most prior research on TET1-based gene regulation has focused on how TET1 affects methylation. However, TET1 can also interact directly with transcription factors and histone modifiers to regulate gene expression. Understanding how TET1 regulates expression to contribute to allergic responses and asthma development thus requires a comprehensive approach. To this end, we measured mRNA expression, DNA methylation, chromatin accessibility and histone modifications in control and TET1 knockdown human bronchial epithelial cells treated or untreated with house dust mite extract.

**Results:**

Throughout our analyses, we detected strong similarities between the effects of *TET1* knockdown alone and the effects of HDM treatment alone. One especially striking pattern was that both *TET1* knockdown and HDM treatment generally led to decreased chromatin accessibility at largely the same genomic loci. Transcription factor enrichment analyses indicated that altered chromatin accessibility following the loss of TET1 may affect, or be affected by, CTCF and CEBP binding. TET1 loss also led to changes in DNA methylation, but these changes were generally in regions where accessibility was not changing.

**Conclusions:**

TET1 regulates gene expression through different mechanisms (DNA methylation and chromatin accessibility) in different parts of the genome in the airway epithelial cells, which mediates inflammatory responses to allergen. Collectively, our data suggest novel molecular mechanisms through which TET1 regulates critical pathways following allergen challenges and contributes to the development of asthma.

## Background

It is estimated that more than 300 million people worldwide suffer from asthma (1), a heterogeneous and complex disease that is treatable but not curable (2). The total cost of asthma in the United States in 2013, including losses due to missed school and work, asthma-related deaths, and medical costs, was $81.9 billion (3). Probably due in part to the heterogeneous nature of the disease, the exact causes for why some individuals develop asthma while others do not are largely unknown. Multiple previous studies have linked epigenetic modifications to asthma prevalence and severity (4–8). In particular, our previous studies indicate that Ten-eleven translocation methylcytosine dioxygenase 1 (TET1) is a key epigenetic modulator in asthma (4, 9). TET1 is a member of the TET enzyme family comprised of three demethylases (TET1, TET2, and TET3). These enzymes convert 5-methyl-cytosine (5mC) to 5-hydroxymethylcytosine (5hmC), then on to 5-formylcytosine (5fC) and 5-carboxylcytosine (5caC) (10). 5fC and 5caC can subsequently be replaced by an unmethylated cytosine via a thymine DNA glycosylase-dependent base excision repair process (11). Methylation of the *TET1* promoter in nasal cells has been associated with severe asthma in children (7). Meanwhile, higher global levels of 5hmC were observed in different tissues from asthmatics (7, 12) and individuals with allergic rhinitis (13). Exposure to house dust mites (HDM) led to a transient increase of *TET1* expression in human bronchial epithelial cells (HBECs) (14), although longer exposure and higher doses may lead to a significant decrease (7). Paradoxically, a study using *TET1* knockout mice and HDM exposure (a well-established model for allergic airway inflammation) indicated that TET1 activity protects against HDM-induced allergic airway inflammation through regulation of interferon signaling and the aryl hydrocarbon receptor pathway (9). *TET1* expression has also been shown to be sensitive to a variety of other stimuli (15), including ethanol exposure (16), repeated cocaine administration (17), ionizing radiation (18), and oxidative stress induced by heavy metals (19).

Prior studies on the association between TET1 and allergic disease and exposures focused primarily on how TET1 regulates DNA methylation. TET1, however, can also impact gene regulation and expression through other mechanisms, such as by interacting with histone modifiers and transcription factors (15, 20, 21). For example, TET1 interacts with hMOF, a histone acetyltransferase that can acetylate H4K16, a mark which generally promotes transcription (15, 22). TET1 has also been shown to bind directly to transcriptional regulators such as EGR-1 (20), SIN3A (21), and ETV2 (23). Although prior studies have separately identified TET1 as a key regulator in allergic airway inflammation and as a regulator of histone modifications, no studies have analyzed the role of TET1 in regulating chromatin accessibility following respiratory challenges.

In this study, we assessed how mRNA expression, DNA methylation, and chromatin accessibility changed in HBECs that had *TET1* knocked down and/or were challenged with HDM. We integrated RNA-sequencing (RNA-seq), whole genome bisulfite sequencing (WGBS), and ATAC-sequencing (ATAC-seq) data, and combined them with computational analyses of transcription factor binding and histone modifications and ChIP-seq analysis of an enhancer-specific histone modification (H3K27ac), to more comprehensively understand the role that TET1 plays in regulating responses to allergic challenges. We found that *TET1* knockdown and HDM treatment both individually had similar effects on gene regulation (especially chromatin accessibility), while combining them causes non-additive effects. Regions that underwent changes in TET1-mediated DNA methylation were generally different physical locations than regions with changes in chromatin accessibility, suggesting separate regulatory mechanisms. These observations provide novel insights into the role of TET1 in the regulation of the epigenetic landscape in airway epithelial cells and may explain contributions of TET1 to asthma.

## Methods

### HBEC growth and TET1 knockdown

HBECs were grown in 1X K-SFM supplemented with EGF, pituitary extract, and pen-strep (complete media). A 12 well plate was seeded with cultured HBEC with near confluent numbers and incubated overnight for attachment. After 24 hours, the complete media was replaced with 1X K-SFM with no serum supplements (minimal media) and incubated for an additional 8 hours. *TET1* was knocked down by adding 30 pmol/mL siRNA (ThermoFisher) and incubated for 24 hours in minimal media. Following control siRNA or *TET1* siRNA incubation, saline or house dust mite (HDM, Greer) was added to HBEC at a concentration of 25μg/mL (25μg dry weight, containing 4.7μg protein, 0.142μg Derp1, 2.15 endotoxin unit, 0.20ng/μL RNA) and incubated for another 24 hours before collection. To summarize, there were four different sample types in our study: 1) wildtype (WT) saline, 2) WT house dust mite (HDM), 3) *TET1* knockdown (KD) saline, and 4) KD HDM.

### ATAC-seq sample processing, library preparation and data analysis

ATAC-seq libraries were prepared for a total of 12 samples (initial n = 3 for WT saline, n = 3 for WT HDM, n = 3 for KD saline, HDM, n = 3 for KD HDM). Samples were prepared according to the Omni-ATAC-seq protocol (modified from (24)). Briefly, 50,000 viable cells were lysed and nuclei were extracted. Nuclei were then treated with transposase at 37°C for 30 minutes (Nx#-tde1, tagment DNA enzyme, Illumina). DNA was extracted using (Zymo DNA Clean and Concentrator-5 Kit) and libraries were prepared. Preamplification with 5 cycles was performed and qPCR was used to determine additional PCR cycles. Libraries were then cleaned up and their quality was determined by bioanalyzer. Indexed libraries were then mixed and sequenced at the DNA technologies core at UC Davis across two sequencing runs. The number of single-end reads per library from samples used in the study ranged from 21–83 million. ATAC-seq data were processed using the ENCODE ATAC-seq pipeline (v 2.0.0) (25, 26) (https://github.com/ENCODE-DCC/atac-seq-pipeline/tree/v2.0.0). Briefly, reads were mapped to hg38 using BowTie2 (27) and peaks were called using MACS2 (28) (https://github.com/macs3-project/MACS/tree/v2.2.4). Based on sample clustering and other QC metrics, two samples from each sample group were selected for further downstream analysis. Principle component analysis and unsupervised clustering were used to evaluate data quality. Differential peaks were identified using MANorm2 (29). We identified differentially accessible peaks (FDR ≤ 0.05, fold-change ≥ 2) in three different comparisons: 1) KD saline vs. WT saline, 2) WT HDM vs. WT saline, and 3) KD HDM vs. WT HDM.

### TF binding analysis

A modified version of HOMER (30) utilizing a log base 2 likelihood scoring system was used to calculate motif enrichment statistics in the differentially accessible chromatin using a large library of human position weight matrix (PWM) binding site models contained in build 2.0 of the CisBP database (31). The RELI method was used to calculate the significance of intersection between the differentially accessible chromatin and publicly available ChIP seq data (~ 10,000 transcription factors) (32).

### RNA-sequencing sample processing and RNA-sequencing data analysis

RNA-seq libraries were prepared for a total of 12 samples across four sample types (n = 4 for WT saline, n = 4 for KD saline, n = 2 for WT HDM, n = 2 for KD HDM). RNA (1μg per sample) was submitted for poly-A RNA library preparation and sequencing at Novogene (Sacramento, CA). Each sample had 21–30 million paired end, 150 base pair reads. Read quality was assessed using FastQC (33). Reads were dynamically trimmed using Trim Galore (34) with the following settings: bases with less than a quality score of 20 were trimmed, 10 bases were trimmed from the 5’ end of both read 1 and read 2, the adapter stringency was set to 6, and reads smaller than 50 bases after trimming were discarded. The reads were aligned to the human transcriptome (hg38) using Bowtie2 (27). Transcripts were subsequently quantified using RSEM (35). The data were then converted into DESeq2 (36) format using tximport (37). We used DESeq2 (36) for differential expression analyses and for hierarchical clustering and principal component analyses. Genes with an absolute shrunken fold change of at least 1.2 and an FDR ≤ 0.05 were considered significantly differentially expressed.

### Chromatin Immunoprecipitation followed by qPCR and sequencing and data analysis

1–2×10^6^ cells were fixed with 0.8% formaldehyde for 3 minutes at RT, and quenched by glycine solution. Cells were lysed with buffer L1 (50nM Hepes pH 8.0, 140nM NaCl, 1mM EDTA, 10% glycerol, 0.5% Igepal CA-630, 0.25% Triton X-100, 1mM Na butyrate) plus protease inhibitor for 10 minutes at 4°C. Nuclei pellets were incubated in buffer L2 (200 mM NaCl, 1 mM EDTA, 0.5 mM EGTA, 10 mM Tris-HCl pH8.0) with protease inhibitor and 1mM Na butyrate. Isolated nuclei were then sonicated in 130ul TE + 0.1% SDS. After sonication, sheared chromatin (0.5–1ug, average length is 200–250bp) was incubated with the appropriate amount of antibody [H3K27ac (Abcam)]. IPed DNA was purified using Qiagen MiniElute Reaction clean-up kit and libraries were made by the UC Davis DNA technologies core using standard TruSeq ChIP Library Preparation Kit (Illumina). ~10 million reads were targeted for each sample. H3K27ac ChIP-Seq was performed for a total of eight samples (2 each from these sample groups: WT saline, KD saline, WT HDM, and KD HDM). We used SciDAP (38) to trim reads (via Trim Galore(34)), align data to the hg38 human reference genome (via Bowtie2 (27)), call peaks (via MACS2 (39)), identify differential H3K27ac presence between sample groups (via DiffBind (40)), and identify super-enhancers within each sample (via ROSE (41, 42)). For the super-enhancer identification, we used a stitching distance of 20 Kb and a TSS exclusion zone of 2.5 Kb. After identifying super-enhancers within each sample, we found consensus super-enhancers for each sample group using BEDtools (43). We first kept only super-enhancers that overlapped with at least one super-enhancer in the other sample from the same group (using BEDtools intersect), then we merged those overlapping super-enhancers together (using BEDtools merge) to form consensus super-enhancers for each sample group. For between sample group comparisons, we used BEDtools intersect to identify super-enhancers that were unique to one sample group vs. another sample group (e.g. KD saline vs. WT saline).

### Whole genome bisulfite sequencing (WGBS) library preparation and data analysis

Whole genome bisulfite sequencing (WGBS) libraries were prepared for a total of 8 samples (2 each from these samples groups: WT saline, KD saline, WT HDM, and KD HDM) using Swift’s Accel-NGS Methyl-Seq Kit. We checked the quality of the libraries using an Agilent 2100 Bioanalyzer, and we measured library concentration using a Qubit high sensitivity DNA assay. Individually barcoded libraries were pooled for sequencing. The pool was then sequenced on one lane of a NovaSeq 6000 S4 flow cell at PE150 at the DNA Technologies and Expression Analysis Cores at the UC Davis Genome Center. After sequencing, reads were demultiplexed using the bcl2fastq Illumina software. The number of paired-end reads per sample ranged from 70–155 million.

We used the CpG_Me pipeline (34, 44–46) to process and align the WGBS data. Because of methylation biases at the 5’ and 3’ ends of reads, we trimmed 10 bases from the 3’ end of both reads, and 10 and 20 bases from the 5’ of read 1 and read 2 respectively using Trim Galore (34). Bismark (45) was then used to align the trimmed reads to the hg38 human reference genome, and to generate CpG count matrices which were used in downstream analyses. MultiQC (46) was used for quality control of the fastq data and the alignments to the reference genome. Following the above data processing, differentially methylated regions (DMRs) were identified for three different comparisons: 1) KD saline vs. WT saline, 2) WT HDM vs. WT saline, and 3) KD HDM vs. WT HDM. We used DMRichR (47–49) to perform these differential methylation analyses. For our differential methylation analyses, we required at least 1x coverage for a CpG for all samples included in a given comparison, a minimum of 5 CpGs for a DMR, 10 permutations for DMR and block analyses, and the single CpG coefficient required to discover testable background regions to be at least 0.05. For a region to be considered a DMR, we required an empirical p-value ≤ 0.01 and a methylation difference of at least 10%.

### Gene ontology, pathway analysis, and significance of overlapping regions

All pathway analyses were performed using IPA (QIAGEN Inc., https://www.qiagenbioinformatics.com/products/ingenuitypathway-analysis ). A cutoff of FDR ≤ 0.05 was applied for significance. Gene set enrichment analysis (GSEA)(50) was used to test gene level associations between changes in gene expression and changes in either methylation or chromatin accessibility. To test whether region-based overlaps were greater than expected by chance, we performed Fisher’s exact tests using BEDtools (43) (a cutoff of p ≤ 0.05 was used to determine significance). We overlapped our significant regions with several datasets from other studies retrieved from the NCBI Gene Expression Omnibus: A549 CTCF binding sites (GSM1003606), A549 CEBPB binding sites (GSM935630), A549 topologically associating domains (GSE92819), and A549 H3K27ac sites (GSE118840). Candidate cis-regulatory elements (cCREs) from A549 were downloaded from https://screen.encodeproject.org/. H3K27ac asthma-associated peaks (51), significant asthma EWAS results (52, 53), differentially methylated positions and regions associated with asthma (54) were also compared to significant regions from our current study. We used the WashU epigenome browser (55) to simultaneously visualize ATAC-seq and WGBS data. For visualization purposes only, ATAC-seq data were normalized between samples based on average transcription start site enrichment.

### IL1B enhancer reporter assay

Human bronchial epithelial cells (HBECs) were grown to 70% confluency in a 96-well plate using keratinocyte serum free medium supplemented with human recombinant Epidermal Growth Factor, Bovine Pituitary Extract (Life Technologies, Calsbad, CA), and 1% Penicillin-Streptomycin antibiotic (Thermo Fisher Scientific, Florence, KY). Twenty-four hours later, starving media (keratinocyte serum free media + 1% pen/strep) was administered. Control plasmid and IL1B enhancer plasmid (100ng/well) were transfected using Lipofectamine 3000 (Thermo Fisher Scientific, Florence, KY), P3000 Reagent (Thermo Fisher Scientific, Florence, KY), and Opti-MEM medium (Thermo Fisher Scientific, Florence, KY) according to the manufacturer’s protocol. Four hours later, the plate was read under fluorescence using a microplate reader (Molecular Devices VERSAmax) at the excitation wavelength of 482 and the emission wavelength of 502. The plasmid was custom-made by Vector Builder Inc. wherein the IL1B enhancer sequence was inserted into a plasmid that contains TurboGFP, a bright green fluorescent protein. After the first fluorescent reading, the plate was placed back in the incubator and was incubated for 24 hours. Then, 25 μg/mL of HDM was added to the appropriate wells. The plate was read again at 4 hours and 24 hours later under fluorescence at the excitation wavelength of 482 and the emission wavelength of 502. For data analysis, the fluorescence readings for the empty wells were averaged and used as the average background. The average background was then subtracted from the fluorescence reading obtained from wells that had samples in them to normalize the data. This analysis was done on the fluorescence readings at 4 hours after plasmid transfection, 4 hours after HDM exposure, and 24 hours after HDM exposure.

## Results

### Loss of TET1 decreases chromatin accessibility in human bronchial epithelial cells.

**Figure 1:**
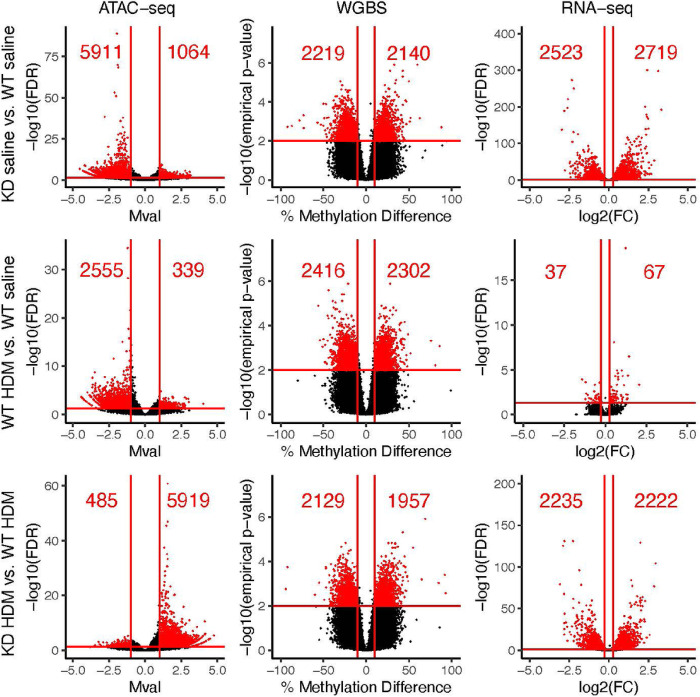
TET1 and HDM-specific chromatin accessibility, DNA methylation, and gene expression. Volcano plots depicting condition-specific changes. Red dots indicate significant differences: For ATAC-seq, differentially accessible peaks required an FDR ≤ 0.05 and a fold change of at least 2. For WGBS, differentially methylated regions required an empirical p-value ≤ 0.01 with at least a 10% difference in methylation percentage. For RNA-seq, differentially expressed genes required an FDR ≤ 0.05 and a fold change of at least 1.2. The red numbers above the plots represent the number of significant results on that side of the plot (*e.g*., 2523 is the number of genes that had decreased expression in KD saline vs. WT saline). Abbreviations: WT saline = *TET1* intact saline samples, WT HDM = *TET1* intact house dust mite samples, KD saline = *TET1* knockdown saline samples, KD HDM = *TET1*knockdown house dust mite samples.

**Figure 2:**
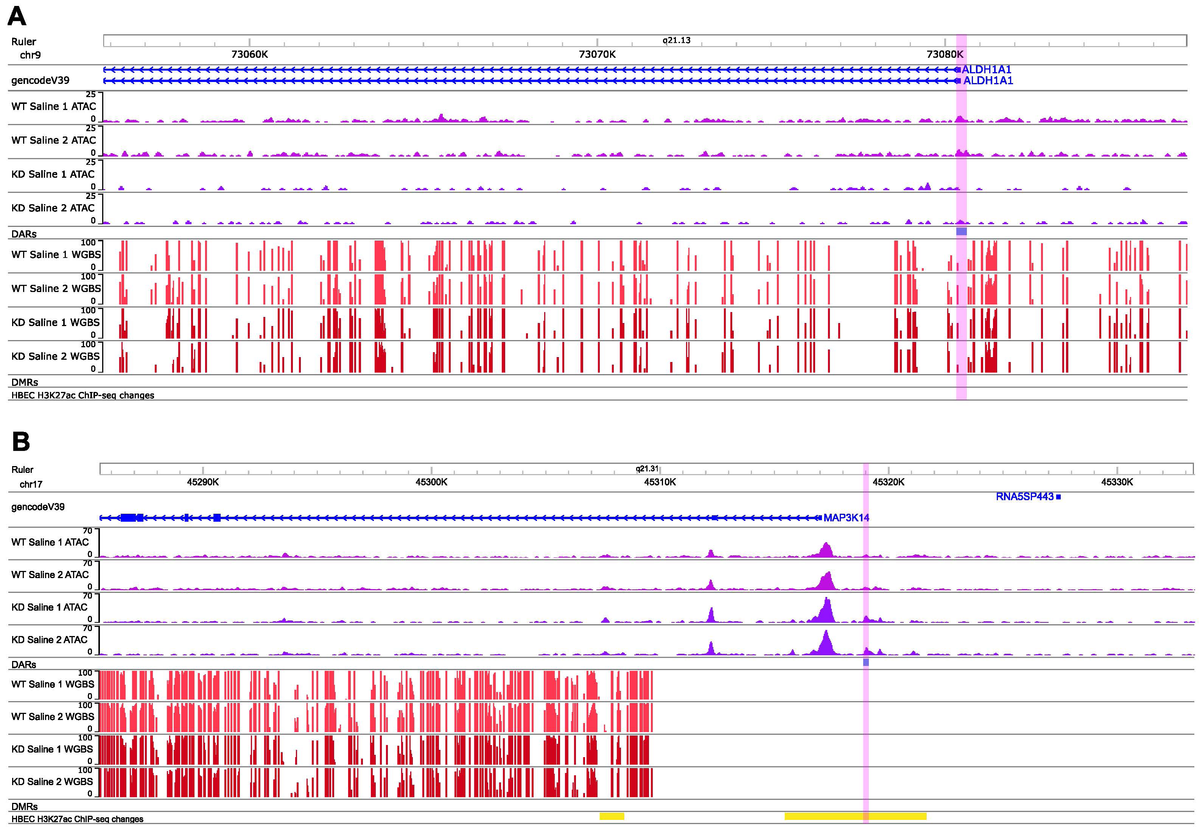
Changes in chromatin accessibility following *TET1*knockdown near genes of interest. Changes in chromatin accessibility in A) *ALDH1A1* (oxidative stress response) and B) *MAP3K14* (Nfkb signaling) following *TET1* knockdown. The peak at *MAP3K14* overlaps with a region also showing differences in H3K27ac ChIP-seq levels. To approximate MANorm2-based data normalization, the ATAC data shown were normalized between samples based on average transcription start site enrichment (for visualization purposes only). The “HBEC H3K27ac ChIP-seq changes” track is from data in our current study. Abbreviations: WT saline = *TET1*intact saline samples, KD saline = *TET1*knockdown saline samples, DARs = Differentially accessible regions, WGBS = Whole genome bisulfite sequencing, DMRs = Differentially methylated regions, HBEC = Human bronchial epithelial cells.

**Figure 3:**
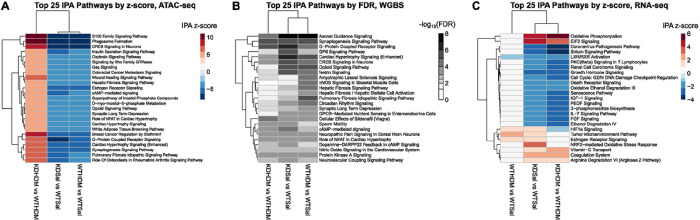
Pathway enrichment of differential ATAC-seq, WGBS, and RNA-seq following *TET1* knockdown and/or HDM treatment. A) Top 25 IPA canonical pathways by total activation z-score from differentially accessible regions in each of the comparisons included in this study (KD saline vs. WT saline, WT HDM vs. WT saline, and KD HDM vs. WT HDM). Activation z-scores were calculated by assuming a positive correlation between chromatin accessibility and gene expression. A red color indicates a positive z-score, indicating that IPA predicted that a given pathway would have increased activity. A blue color indicates a negative z-score, indicating that IPA predicted that a given pathway would have decreased activity. B) Top 25 IPA canonical pathways by total false discovery rate (FDR) from differentially methylated regions in each of the comparisons included in this study. Given the context dependent relationship between methylation and expression, activation z-scores could not reliably be calculated for the methylation data. C) Top 25 IPA canonical pathways by total activation z-score from differentially expressed genes in each of the comparisons included in this study. Abbreviations: WT saline = *TET1*intact saline samples, WT HDM = *TET1*intact house dust mite samples, KD saline = *TET1*knockdown saline samples, KD HDM = *TET1*knockdown house dust mite samples.

In order to understand how TET1 regulates gene expression in HBECs, we knocked down *TET1* using siRNA (48 hour incubation led to ~ 72% downregulation, Supplementary Fig. 1) and examined the impact on chromatin accessibility using ATAC-seq. Compared to control WT saline cells, cells with deficient TET1 expression (KD saline) showed a general decrease in chromatin accessibility (Fig. 1). Overall, we identified 6975 differentially accessible peaks (FDR ≤ 0.05, fold-change ≥ 2) in the KD saline vs. WT saline comparison, which were annotated to 4669 unique genes (Supplementary Table 1, selected candidates shown in Fig. 2 and Supplementary Fig. 2). The majority of these peaks (84.7%) were less accessible in KD saline cells. IPA analysis on the genes associated with significantly differentially accessible ATAC-seq peaks revealed 111 significantly enriched canonical pathways (FDR ≤ 0.05; Supplementary Table 2, top pathways by cumulative absolute activation z-score shown in Fig. 3A). Some enriched pathways of interest included Leukocyte extravasation signaling, IL-15 production, and Pulmonary Fibrosis Idiopathic Signaling Pathway. Genes involved in oxidative stress response (*e.g. TLR3, TLR5, TLR10, ALDH1A1, ALDH1B1, ALDH2*, and *ALDH7A1*), type I immune response (*e.g*. *STAT3, STAT4, STAT5B, IFNGR1,* and *OAS1*), type II immune response (*e.g. STAT3, STAT4,* and *STAT5B*) and type 17 immune response (*e.g. AHR, AHRR,* and *IRF4*) all showed changes in accessibility (mostly reduced) when TET1 was low (Supplementary Table 1). Together, these data show that TET1 promotes chromatin accessibility in HBECs.

### Loss of TET1 leads to changes in methylation, primarily in regions where chromatin accessibility did not change.

**Figure 4:**
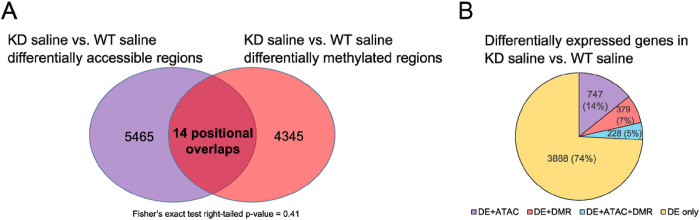
Changes in DNA methylation and chromatin accessibility affect different regions of the genome following *TET1* knockdown. A) Venn diagram showing positional overlaps between regions changing in accessibility and DNA methylation in KD saline vs. WT saline. There were not more overlaps than expected by chance (p = 0.41). B) Pie chart showing overlaps at the gene level for differential expression (DE), differentially accessible regions (ATAC), and differentially methylated regions (DMRs). Abbreviations: WT saline = *TET1* intact saline samples, KD saline = *TET1* knockdown saline samples, DE = differentially expressed, ATAC = differentially accessible regions, DMRs = differentially methylated regions.

It is well established that TET1 regulates DNA methylation (56), and that DNA methylation has a direct impact on transcription factor binding (57, 58). Some evidence suggests that DNA methylation may directly influence chromatin accessibility as well (59). Therefore, we performed genome-wide bisulfite sequencing to evaluate the DNA methylation landscape in *TET1*-deficient HBECs. Given that TET1 is a demethylase, we expected that *TET1* knock down samples would have generally higher methylation levels than samples where *TET1* was intact. However, this was not the case. When comparing *TET1* KD saline samples to WT saline samples, we identified 4359 significant DMRs (empirical p-value ≤ 0.01, ≥10% difference) annotated to 3224 unique genes (Supplementary Table 3). Of these DMRs following *TET1* knockdown, 49.1% were hypermethylated (Fig. 1). These DMR-associated genes were enriched for 94 IPA pathways (Supplementary Table 4, top pathways in Fig. 3B). Enriched pathways of interest include Pulmonary Fibrosis Idiopathic Signaling Pathway, IL-8 Signaling, and IL-15 Production. Out of these 4359 DMRs between KD saline and WT saline, only 14 had overlapping coordinates with one of the 5479 differentially accessible regions between these two sample groups (Fig. 4A). Collectively, although WGBS measures the sum of 5mC and 5hmC and may miss regions that undergo simultaneous and similar opposite changes in both marks (60), these data suggest that TET1 independently influences chromatin accessibility and DNA methylation in HBECs.

### TET1 knockdown-induced chromatin accessibility and methylation changes influence gene expression.

**Figure 5:**
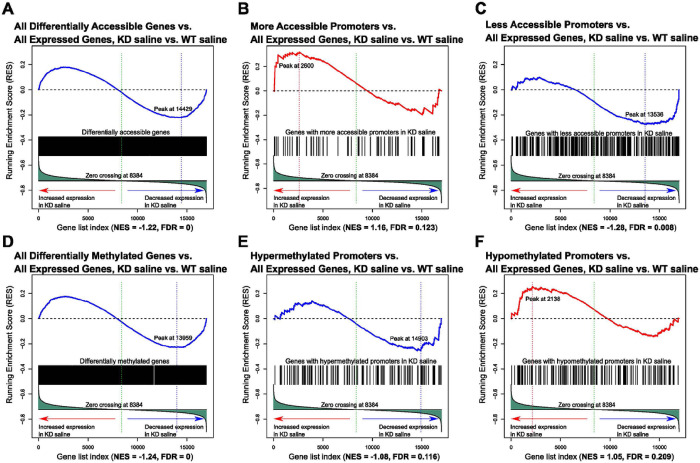
Gene set enrichment analyses (GSEA) reveal strong correlation between changes in accessibility or DNA methylation and changes in gene expression. The top of each plot shows the running enrichment score, where a positive value indicates an enrichment for changes in either accessibility or methylation in genes that are increasing in gene expression, while a negative value indicates an enrichment for changes in either accessibility or methylation in genes that are decreasing in gene expression. The enrichment score (ES) is the point of the largest deviation from zero in the upper portion of the plot. The normalized enrichment score (NES) can be compared across gene sets. The FDR for the enrichment (based on a comparison with the gene set null distribution) is also shown. The following sets of genes were used as input to compare against gene expression changes (KD saline vs. WT saline comparison): A) all changes in accessibility, B) increased accessibility in promoters, C) decreased accessibility in promoters, D) all changes in methylation, E) increased methylation in promoters, F) decreased methylation in promoters. Abbreviations: WT saline = *TET1* intact saline samples, KD saline = *TET1* knockdown saline samples.

We next performed RNA-sequencing to assess the effect of *TET1* knockdown on gene expression and to attempt to correlate changes in gene expression with changes in chromatin accessibility and/or DNA methylation. We identified 5242 genes that were differentially expressed in the KD saline vs. WT saline comparison (Supplementary Table 5). There was a fairly even split of upregulation and downregulation in these DE genes (51.9% upregulated, Fig. 1). These differentially expressed genes were enriched for IPA pathways of interest such as IL-8 Signaling, HIF1α Signaling, Aryl Hydrocarbon Receptor Signaling, and NRF2-mediated Oxidative Stress Response (Supplementary Table 6, top pathways in Fig. 3C). Importantly, these changes in gene expression correlated with changes in DNA methylation and chromatin accessibility following *TET1* knockdown. Genes near regions with changes in accessibility tended to have reduced expression in KD saline (Fig. 5A). Genes with more accessible promoters in KD saline showed a trend for increased expression (not significant, Fig. 5B), while genes with less accessible promoters were significantly more likely to have decreased gene expression (Fig. 5C). Genes near DMRs were significantly more likely to be downregulated in KD saline (Fig. 5D). Genes with hypermethylated promoters in KD saline showed a trend of having decreased expression (Fig. 5E), while genes with hypomethylated promoters showed a trend of having increased expression (Fig. 5F).

To further compare the effects of methylation and accessibility on gene expression, we also performed IPA pathway analyses on genes that were both associated with a DMR and differentially expressed following *TET1* knockdown (but not differentially accessible, DMR + DE only). These genes (7.2% of all DE genes, Fig. 4B) were enriched for 9 IPA canonical pathways (Supplementary Table 7). We compared these pathways to enriched pathways in genes that were simultaneously associated with a differentially accessible peak and differentially expressed, but not differentially methylated (ATAC + DE only). These genes (14.3% of all DE genes, Fig. 4B) were enriched for 132 IPA canonical pathways (Supplementary Table 7). Out of the nine enriched pathways from the DMR + DE only set, seven were also enriched in the ATAC + DE only set. On the other hand, some of the 126 pathways enriched in ATAC + DE only but not DMR + DE only include EIF2 Signaling, IL-8 Signaling, NRF2-mediated Oxidative Stress Response, and Xenobiotic Metabolism Signaling. There were also 228 genes (4.4% of all DE genes, Fig. 4B) that were simultaneously DE, differentially methylated, and differentially accessible (ATAC + DMR + DE). There was a physical overlap between the DMR and the differentially accessible region in only 14 of these genes. The ATAC + DMR + DE genes were only enriched for one IPA canonical pathway (Protein Kinase A Signaling), and this pathway was not enriched in either the ATAC + DE only or DMR + DE only groups. Overall, these results imply that changes in chromatin accessibility and DNA methylation following *TET1* knockdown both impact gene expression, but they often affect different genes, pathways, and regions of the genome.

### HDM challenge closes chromatin in a similar fashion to TET1 knockdown in HBECs.

**Figure 6:**
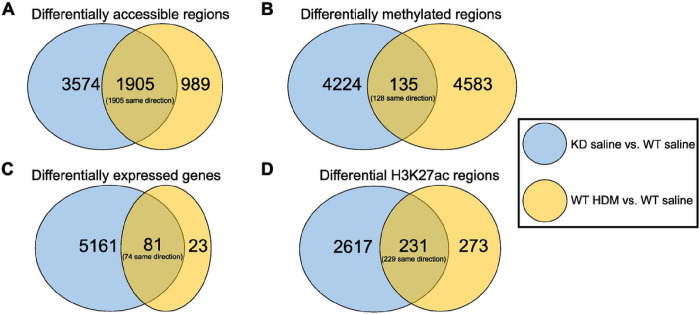
Overlap between changes following *TET1* knockdown and changes following HDM treatment. Venn diagrams depicting the overlaps between changes in the KD saline vs. WT saline and the WT HDM vs. WT saline comparisons in A) differentially accessible regions, B) differentially methylation regions, C) differentially expressed genes, and D) differential H3K27ac regions. Changes in the “same direction” refer to regions/genes that either increased in both comparisons or decreased in both comparisons. Abbreviations: WT saline = *TET1* intact saline samples, WT HDM = *TET1* intact house dust mite samples, KD saline = *TET1* knockdown saline samples, KD HDM = *TET1* knockdown house dust mite samples.

HDM promotes allergy and asthma in humans (61, 62). We previously found that loss of TET1 exaggerated HDM-induced allergic airway inflammation in mice (9). In our current experiments, HDM significantly downregulated Tet1 expression following 24hrs of challenge (Supplementary Fig. 1). Therefore, we hypothesized that TET1 loss and HDM treatment have a similar impact on chromatin accessibility. We observed 2894 ATAC-seq peaks that were significantly different between WT HDM and WT saline cells (FDR ≤ 0.05, fold-change ≥ 2; Fig. 1, Supplementary Table 1, annotated to 2328 genes). Similar to the changes we observed to chromatin accessibility following *TET1* knockdown, HDM treatment primarily led to decreased chromatin accessibility (88.3%) in regions where accessibility was altered. 65.8% of these peaks overlapped with a significant peak from the KD saline vs. WT saline comparison, significantly more than expected by chance (p ~ = 0, Fig. 6A). Even more striking is that for each overlap, the direction of change in the HDM-treated group was the same as the direction of change in the KD saline group (*i.e*. if accessibility increased in KD saline vs. WT saline, it also increased in WT HDM vs. WT saline). A principal component analysis on the chromatin accessibility data revealed that WT HDM samples clustered closest with KD saline samples, with these two groups split from WT saline and KD HDM along PC1 (Supplementary Fig. 3). Genes associated with differentially accessible peaks were significantly enriched for 88 IPA pathways (Fig. 3A, Supplementary Table 2). The top five most enriched pathways in genes associated with differentially accessible peaks following HDM treatment were all in the top 32 most enriched pathways following *TET1* knockdown. Additionally, all five of these pathways had the same inferred direction of change. 65 (73.0%) enriched pathways following HDM treatment were also enriched following *TET1* knockdown, including TGF-β Signaling and Pulmonary Fibrosis Idiopathic Signaling Pathway (Supplementary Table 2). Of these 65, 55 had similar z-scores (*i.e*. the inferred direction of change in activation was the same), 8 had no activation z-scores calculated (due to pathway limitations), and 2 had opposing activation z-scores. Collectively, our data show that HDM challenge and TET1 deficiency have similar impacts on chromatin accessibility in airway epithelial cells, with both likely impacting proinflammatory pathways and lung function.

### HDM treatment also alters DNA methylation patterns in a similar fashion to TET1 knockdown in HBECs.

We previously showed that HDM challenges led to changes in 5mC and 5hmC in HBECs using microarrays, and changes in 5mC responsive to HDM and diesel particles significantly correlated with changes in gene expression (14). When we compared methylation patterns between WT HDM HBECs and WT saline HBECs using WGBS, we identified 4718 significant DMRs (empirical p-value ≤ 0.01, at least 10% difference in methylation) annotated to 3428 unique genes (Supplementary Table 3). Of these DMRs, 48.8% were hypermethylated in WT HDM compared to WT saline (Fig. 1). There were significantly more positional overlaps than expected between DMRs in the KD saline vs. WT saline comparison and DMRs in the WT HDM vs. WT saline comparison (p = 1.2 × 10^−92^, Fig. 6B). For these positional overlaps, methylation changed in the same way (*i.e*. increased or decreased) after either *TET1* knockdown or HDM treatment 94.8% of the time. These positional overlaps were annotated to genes such as *CLU, TLR7,* and *IL13RA1* (Supplementary Table 8). At the gene level, out of the genes that were associated with DMRs following TET1 knockdown, 36.7% were also associated with at least one DMR following HDM treatment. The DMR-associated genes following HDM treatment were enriched for 70 IPA canonical pathways (Supplementary Table 4). These pathways were generally similar to enriched pathways from DMR-associated genes in the KD saline vs. WT saline comparison; there were six overlapping pathways in the top ten enriched pathways from each comparison. Out of the enriched pathways from DMR-associated genes following *TET1* knockdown, 59.6% were also enriched in DMR-associated genes following HDM treatment, including Pulmonary Fibrosis Idiopathic Signaling Pathway, Nitric Oxide Signaling in the Cardiovascular System, Leukocyte Extravasation Signaling, and IL-8 Signaling. Overall, HDM treatment and *TET1* knockdown had similar effects on the methylome, potentially affecting genes and pathways contributing to inflammation and lung function.

### HDM treatment led to few changes in expression, but these changes were similar to changes following TET1 knockdown.

We next performed RNA-sequencing to assess the effect of HDM treatment on gene expression, and to compare these results with how *TET1* knockdown affected gene expression. We identified 104 DE genes in the WT HDM vs. WT saline comparison (Supplementary Table 5). There were many fewer DE genes following HDM treatment alone compared to after *TET1* knockdown alone (104 vs. 5242 genes). Of these DE genes, 64.4% were upregulated following HDM treatment. Out of these DE genes, 77.9% were also DE in the KD saline vs. WT saline comparison, indicating that most genes with changed expression following HDM treatment were also affected by *TET1* knockdown (Fig. 6C). Further supporting this notion was the finding that out of these shared DE genes, 91.4% changed in the same direction (*i.e*. HDM treatment and *TET1* knockdown both led to downregulation or both led to upregulation). These genes whose expression was altered similarly following HDM treatment or *TET1* knockdown included *IL1A* (increased), *IL1B* (increased), *IFNGR2* (increased), and *AHR* (decreased) (Supplementary Fig. 4A). We also performed IPA pathway analysis on the DE genes from the WT HDM vs. WT saline comparison to see if expression levels in similar pathways were affected by both HDM treatment and *TET1* knockdown (Fig. 3C, Supplementary Table 6). Of 22 enriched IPA pathways, 11 (50%) were also enriched in DE genes from the KD saline vs. WT saline comparison. Out of these 11 shared pathways, 7 had estimated activation z-scores in the WT HDM vs. WT saline comparison (Supplementary Fig. 4B); in all 7 cases, the activation z-scores for both comparisons indicated consistent pathway level changes. For example, the activation z-scores for Aryl Hydrocarbon Receptor Signaling were negative in both cases, indicating that expression changes following HDM treatment alone and *TET1* knockdown alone both likely led to some deactivation of that pathway. Overall, despite HDM treatment leading to many fewer DE genes than knocking down *TET1* in HBECs, genes and pathways that were affected by HDM treatment were frequently also affected by *TET1* knockdown as well.

### Loss of TET1 results in a transcriptomic signature enriched in stress and immune response following HDM challenges in HBECs.

As our data demonstrated that TET1 loss tends to have a similar impact to HDM challenge on chromatin accessibility, DNA methylation and gene expression, we next compared expression in KD HDM HBECs to WT HDM HBECs through RNA-seq analysis. In a pairwise analysis, we identified 4457 genes that were differentially expressed (FDR ≤ 0.05, FC ≥ 1.2, Fig. 1, Supplementary Table 5). Of these DE genes, 49.9% were significantly upregulated in KD HDM compared to WT HDM HEBCs (Fig. 1). The majority of these genes (67.6%) were also differentially expressed when comparing KD saline vs. WT saline. Out of the overlapping DE genes, 99% showed changes in the same direction when *TET1* was knocked down, suggesting that these gene expression changes were primarily driven by TET1 loss regardless of the presence of HDM (Supplementary Fig. 3). Pathway analysis via IPA on all DE genes from the KD HDM vs. WT HDM comparison (Fig. 3C, Supplementary Table 6) revealed the enrichment of 269 IPA pathways. These genes were enriched for pathways of interest such as Oxidative Phosphorylation, Mitochondrial Dysfunction, HIF1α signaling, NRF2-mediated Oxidative Stress Response, and Aryl Hydrocarbon Receptor Signaling. Additionally, the significant enrichment and strong activation (activation z-score = 3.97) of EIF2 Signaling in DE genes in KD HDM vs. WT HDM HBECs suggests an upregulation of protein translation. Interestingly, we also observed significant enrichment for genes involved in the regulation of cell cycle and division (Cell Cycle: G2/M DNA Damage Checkpoint Regulation, Cyclins and Cell Cycle Regulation, Cell Cycle: G1/S Checkpoint Regulation, etc.). Significant downregulation was observed in several cyclin and cyclin-dependent kinases (CCND1, CCND3, CDC25A and CDK4/6) and transcriptional regulators required for E2F-mediated transcription (E2F6), which may block G1/S cycle progression, and this is similar to what we observed in KD saline compared to WT saline.

To further elucidate the role of TET1 in regulating responses to HDM, we separately analyzed the 1444 genes that were DE in KD HDM vs. WT HDM but not in KD saline vs. WT saline. There were several DE genes in this set that are subunits of the main complexes in the electron transport chain, including *ATP5F1C, ATP5MC1, COX5A, NDUFA4, NDUFA7, UQCR11,* and *UQCRQ*. There were also immune and detoxification genes such as *ARNT, FLOT2, GSTO2, JUN, JUNB, IL17RE, IRF2BP2, SMAD4, TLR5,* and *TRAF4* (examples shown in Supplementary Fig. 5). These genes were enriched for seven IPA pathways, including Oxidative Phosphorylation, Mitochondrial Dysfunction, and Autophagy (Supplementary Table 9). All of these pathways were also enriched in the KD saline vs. WT saline DE genes (Supplementary Table 6) even though overlapping genes were specifically filtered out for this analysis, indicating that TET1 broadly regulates these pathways but that the specific genes that are impacted by the loss of TET1 are at least partially context-dependent.

### HDM influences the regulation of chromatin accessibility and DNA methylation by TET1.

In contrast to the mostly reduced accessibility observed in KD saline cells compare to WT saline cells, we observed a strong pattern of increased accessibility in KD HDM cells compared to WT HDM (Fig. 1). There were 6404 significantly different ATAC-seq peaks (Supplementary Table 1, annotated to 4449 unique genes), and 92.4% of the peaks showed increased peak intensity in KD HDM (Fig. 1). Of these genes associated with differential chromatin accessibility in KD HDM vs. WT HDM, 51.8% were also associated with differential accessibility in the KD saline vs. WT saline comparison. Only 10.5% of these genes overlapping genes, however, showed consistent changes in direction in the two comparisons (*i.e*. a gene increasing in accessibility in KD HDM vs. WT HDM and also increasing in KD saline vs. WT saline). Out of the differentially accessible peaks in KD HDM vs. WT HDM, 30.8% overlapped positionally with a differentially accessible peak in KD saline vs. WT saline (p ~ = 0). For these positional overlaps, in only 1.4% did *TET1* knockdown have a consistent effect of either increasing or decreasing accessibility regardless of whether HDM was present or not. These results indicate a likely interaction between *TET1* knockdown and the presence of HDM since the effect of *TET1* knockdown was different depending on the environment. This was further supported by a principal component analysis where PC1 (which explained 65.8% of the variance) separated WT saline and KD HDM samples from WT HDM and KD saline samples (Supplementary Fig. 3). Pathway analysis via IPA on genes associated with changes in accessibility revealed significant enrichment for 112 pathways (Supplementary Table 2), 61.6% of which also were enriched in significant ATAC peaks from KD saline vs. WT Saline. Of the pathways where an activation z-score could be assigned (assuming a positive correlation between chromatin accessibility and gene expression), 96.8% showed opposite patterns of activation in KD HDM vs. WT HDM and KD saline vs. WT saline (Supplementary Fig. 4C). This indicates that knocking down *TET1* consistently leads to changes in chromatin accessibility in certain pathways, but that precisely how accessibility is affected depends on whether HDM is present or not.

When we compared genome-wide DNA methylation levels in KD HDM samples to WT HDM samples, we identified 4086 DMRs (Fig. 1) annotated to 3037 unique genes (Supplementary Table 3). The 3037 DMR-associated genes were enriched for 68 IPA pathways (Fig. 3B, Supplementary Table 4). Of the genes that were associated with a DMR in this comparison (KD HDM vs. WT HDM), 34.4% were also DMR-associated in the KD saline vs. WT saline comparison. Of these overlapping genes, the average methylation changes were in the same direction following *TET1* knockdown 49.4% of the time. There were 44 positional overlaps between KD HDM vs. WT HDM DMRs and KD saline vs. WT saline DMRs (more than expected by chance, p = 6.5 × 10^−15^). There were consistent directional changes in methylation regardless of whether HDM was present in 43.2% of these positional overlaps. These results imply that, similar to chromatin accessibility, methylation changes following TET1 loss were influenced by the presence of HDM. There were only 10 positional overlaps between KD HDM vs. WT HDM DMRs and differentially accessible regions, adding another piece of evidence that the changes we observed in chromatin accessibility following *TET1* knockdown generally happened in different regions of the genome than changes in DNA methylation.

### Loss of TET1 and HDM treatment lead to similar changes in H3K27ac, and these changes are enriched for asthma-associated changes in H3K27ac.

**Figure 7:**
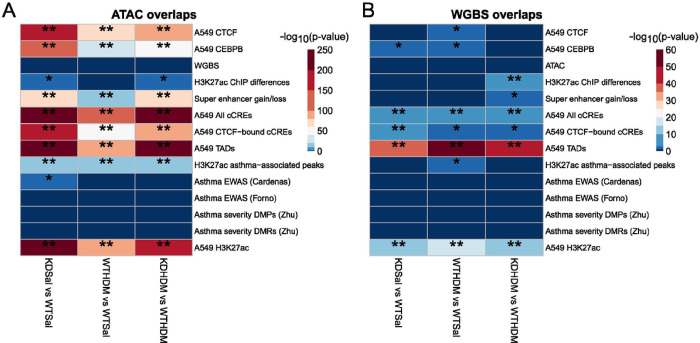
Overlap significance between our data and public datasets of interest. Significance of overlaps between various genomic regions of interest with our A) ATAC-seq (chromatin accessibility) peaks and our B) WGBS (methylation) DMRs. The following labels refer to data from the current study: “WGBS”, “ATAC”, “H3K27ac ChIP-differences”, and “Super-enhancer gain/loss”. “A549 CTCF” (GSM1003606), “A549 CEBPB” (GSM935630), “A549 TADs” (GSE92819), and “A549 H3K27ac” (GSE118840) refer to data retrieved from the NCBI Gene Expression Omnibus (GEO). “A549 All cCREs” and “A549 CTCF-bound cCREs” refer to candidate cis-regulatory elements downloaded from https://screen.encodeproject.org/. The rest of the data were retrieved from the following sources (first author’s last name is in parentheses): “H3K27ac asthma-associated peaks (McErlean)”(51), “Asthma EWAS (Cardenas)”(52), “Asthma EWAS (Forno)”(53), “Asthma severity DMPs (Zhu)”(54), and “Asthma severity DMRs (Zhu)”(54). * = significant by p-value (< 0.05), ** = significant by Bonferroni corrected p-value (< 0.0012). Abbreviations: WT saline = *TET1* intact saline samples, WT HDM = *TET1* intact house dust mite samples, KD saline = *TET1* knockdown saline samples, KD HDM = *TET1* knockdown house dust mite samples

Since H3K27ac is highly enriched at active enhancers (63), we performed H3K27ac ChIP-seq and searched for regions with changes in H3K27ac signal intensity. We identified 2848 differential regions in which KD saline and WT saline groups had similar signal strength, with only 16 regions showing increased H3K27ac in KD saline and 2832 regions showing decreased H3K27ac in KD saline (Supplementary Table 10). Out of these 2848 regions, 34.2% were annotated to a differentially expressed gene, of which 57.1% showed the generally expected positive correlation between H3K27ac levels and expression (enriched pathways in Supplementary Table 11). Interestingly, 33 of these differences overlapped positionally with changes in chromatin accessibility (p = 0.03, Fig. 7A), including an overlap at *MAP3K14* (Fig. 2B), which stimulates NFKB signaling (64).

As enhancers may cluster together and form super-enhancers to drive transcription of genes implicated in cell identity (65, 66) and disease (51, 67), we next compared the presence of super-enhancers in KD saline vs. WT saline samples. We identified 109 consensus “gained” super-enhancers in KD saline samples that did not overlap with any consensus super-enhancers in WT saline samples, and 329 “lost” consensus super-enhancers in WT saline samples with no overlaps in the KD saline group (Supplementary Table 12). These 438 “gained” or “lost” super-enhancer regions overlapped with 140 DE genes (*i.e*. the closest gene to the super-enhancer was DE) and 146 overlapped physically with regions with changes in chromatin accessibility (p = 1.75×10^−62^, Fig. 7A). A positive correlation between “gain” or “loss” of a nearby super-enhancer and changes in expression might be generally expected, but we observed this expected correlation only 41.4% of the time. Overall, we generally observed decreased H3K27ac signal in KD saline samples compared to WT saline samples, but these changes were not well correlated with changes in gene expression, possibly because other regulatory changes were underlying gene expression changes, changes in expression came after the time point that we sampled, or our “nearest gene” annotation strategy might not always connect a regulatory region to its affected gene.

Similar to what we observed following *TET1* knockdown, a majority of regions with significant differences in H3K27ac showed decreased levels in WT HDM compared to WT saline (374/504, 74.2%) (Supplementary Table 10). These changes in H3K27ac overlapped with 9 DE genes, and there was a positive correlation between changes in H3K27ac and gene expression in 7 of these instances (77.8%). These regions largely overlapped with differential H3K27ac regions from the KDSal vs. WTSal comparison (Fig. 6D) Also similar to what we observed following *TET1* knockdown, there were many more “lost” super-enhancers in WT HDM (329) than gained super-enhancers (86) in WT HDM (Supplementary Table 12). These 415 “gained” or “lost” super-enhancer regions overlapped with only 4 DE genes (there were only 104 DE genes in WT HDM vs. WT saline in total), and in only 1 out of 4 of these DE genes did we observe a positive correlation between super-enhancer presence and expression changes. When comparing KD HDM to WT HDM, out of 1479 regions showing significant changes in H3K27ac in regions where both groups had H3K27ac signal, 99.4% showed decreased levels of H3K27ac in KD HDM (Supplementary Table 10). There were 482 of these regions with significant differences in H3K27ac that overlapped with a significant DE gene, and 67% showed a positive correlation with changes in expression. However, unlike what we observed following *TET1* knockdown and HDM treatment alone (both led to many more “lost” super-enhancers based on analysis of H3K27ac ChIP-seq), the number of “lost” super-enhancers (194) was similar to the number of “gained” super-enhancers (226) in KD HDM (Supplementary Table 12). These 420 “gained” or “lost” super-enhancer regions overlapped with 116 DE genes (there were only 104 DE genes in WT HDM vs. WT saline in total), but only 47.4% of the time did we observe a positive correlation between super-enhancer presence and expression changes. These super-enhancer results provide another example of the combination of TET1 knockdown and HDM treatment having an interactive effect (rather than additive) on epigenomic regulation. Similar to the results from KD saline vs. WT saline, there was only limited correlation between super-enhancer gain or loss and expression, but there was some correlation between changes in H3K27ac levels and expression. Overall, this indicates that *TET1* knockdown and HDM treatment both lead to similar decreases in H3K27ac presence, again highlighting that these two conditions have a similar impact on the overall regulatory landscape.

Besides enhancer-specific markers, we also observed significant overlap between TET1-regulated differentially accessible regions and all A549 ENCODE (25) predicted cCREs (candidate cis-regulatory elements, defined by chromatin accessibility, CTCF binding, H3K27ac and H3K4me3) and CTCF-bound cCREs (Fig. 7A). As CTCF is important in setting up 3-D chromatin interactions and topologically associating domains (TADs), we also quantified the overlap between differentially accessible regions and TADs in A549 cells (GSE92819) and found significant overlap (Fig. 7A). In addition, TET1 regulates the expression of genes involved in asthma to protect against allergic asthma (9, 68). Therefore, we searched for the presence of asthma-associated variations in DNA methylation and histone acetylation found in airway epithelial cells among TET1-loss induced differentially accessible regions and DMRs. We observed significant enrichment for asthma-associated H3K27ac changes (51), but not asthma-associated DMRs or DMPs (52–54) (Fig. 7A and 7B). Therefore, our data support that TET1-mediated chromatin accessibility might influence H3K27ac marks and cis-regulatory elements and CTCF binding to regulate gene expression, which may explain its role in protection from inflammation and asthma.

### Altered chromatin accessibility and DNA methylation following loss of TET1 and HDM treatment may affect CTCF and CEBP binding.

**Figure 8:**
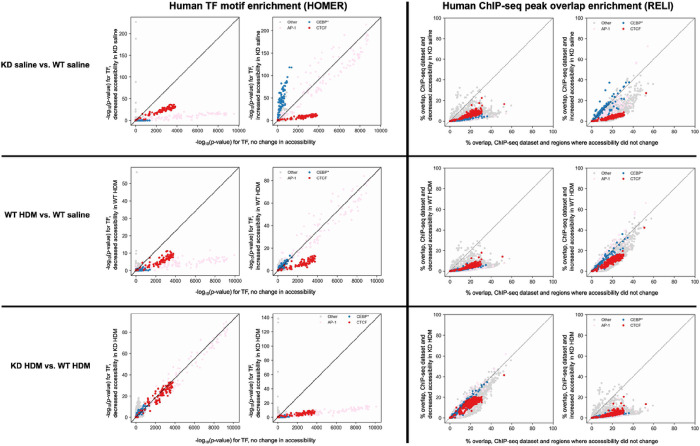
Enrichment of transcription factor (TF) binding sites in differentially accessible ATAC-seq peaks. Motif enrichment (HOMER) results were generated by comparing differentially accessible ATAC-seq peaks using a custom HOMER database of transcription factor binding motifs obtained from the Cis-BP database (see Methods). The ChIP-seq peak enrichment results (RELI) were generated by comparing differentially accessible ATAC-seq peaks with public ChIP-seq peaks monitoring the binding of transcriptional regulators. Y-axes show the relative enrichment for a given ChIP-seq dataset (RELI panels) or TF binding motif (HOMER panels) in regions changing in accessibility (*i.e.* differential ATAC-seq peaks). X-axes show the relative enrichment for regions not changing in accessibility (*i.e*. statistically equivalent ATAC-seq peaks). Points straying from the diagonal line indicate a difference in enrichment between these sets of regions. Abbreviations: WT saline = *TET1* intact saline samples, WT HDM = *TET1* intact house dust mite samples, KD saline = *TET1* knockdown saline samples, KD HDM = *TET1* knockdown house dust mite samples.

As altered chromatin accessibility may impact the binding of TFs and therefore lead to changes in gene expression, we next performed unbiased HOMER and RELI transcription factor enrichment analyses. Using a custom HOMER database of all human transcription factor binding motifs obtained from the CisBP database (31) (see Methods), we compared TF binding motif enrichment in peaks that had changed accessibility following *TET1* KD with peaks that did not show any significant change in accessibility (Fig. 8). We also performed a similar analysis with RELI, which utilizes results from publicly available ChIP-seq experiments instead of TF binding motifs (Fig. 8). In regions where KD saline had reduced accessibility, both analyses indicated lower enrichment of CTCF and CEBP binding sites compared to regions that were not changing in accessibility (Fig. 8). In regions where KD saline had increased accessibility, however, both analyses showed increased enrichment of CEBP binding sites and decreased enrichment of CTCF binding sites. These data suggest the *TET1*-regulated chromatin accessibility may specifically impact CTCF and CEBP binding. In contrast, we observed no enrichment for either CTCF or CEBP binding sites within DMRs (Supplementary Fig. 6).

In order to further test the association between CTCF and CEBP binding and changes in chromatin accessibility, we compared binding sites for CTCF (GSM1003606) and CEBPB (GSM935630) in A549 cells to our differentially accessible peaks. The differentially accessible peaks overlapped much more frequently than expected by chance with the CTCF peaks (Fig. 7A). DMRs between KD saline HBECs and WT saline HBECs, however, did not overlap more frequently than expected with the CTCF binding sites from A549 cells (Fig. 7B), emphasizing how the loss of TET1 likely affects gene regulation in different ways in different types of genomic environment. There was also significant overlap between both differentially accessible peaks (Fig. 7A) and DMRs (Fig. 7B) and CEBPB binding sites in A549 cells. The much lower p-value and much greater odds ratio for the differentially accessible peaks, however, imply a more significant overlap between differentially accessible peaks and CEBPB sites than between DMRs and CEBPB sites. Overall, TET1 loss-associated changes in accessibility were clearly enriched for both CTCF and CEBPB motifs and ChiP-seq peaks, while changes in methylation showed some evidence for enrichment of CEBPB ChIP-seq peaks.

Similarly, TF motif enrichment analysis (HOMER) revealed that in peaks where HDM treatment led to increased accessibility, there was more enrichment for CEBP binding sites and fewer CTCF binding sites than in peaks where there were no changes in accessibility (Fig. 8). In peaks where HDM treatment led to reduced accessibility, both CTCF and CEBP binding sites were less than expected (Fig. 8). These HOMER results were very similar to the HOMER results from the KD saline vs. WT saline comparison, implying that chromatin changes caused by HDM treatment and *TET1* knockdown have similar impacts on TF binding. The RELI analysis (which compares significant results to publicly available ChIP-seq studies rather than searching for TF binding motifs) showed similar enrichment patterns as the HOMER analysis (Fig. 8). Despite observing opposing changes in accessibility, similar to the results when *TET1* was knocked down in saline, we observed less overlap with CTCF binding sites than expected in regions with increased accessibility in KD HDM (Fig. 8). Together, these results support that altered chromatin accessibility and DNA methylation following loss of TET1 and HDM treatment may alter the binding of CTCF and CEBP.

### A TET1-mediated regulatory element at the IL1B locus enhances gene expression to the same extent as HDM challenge.

**Figure 9:**
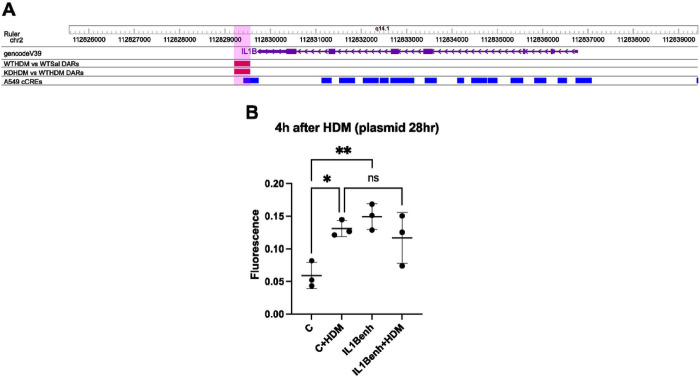
Changes in chromatin accessibility in the *IL*1Blocus following HDM treatment correspond with expression changes following HDM and/or *IL1B* enhancer treatment. A) Changes in chromatin accessibility following HDM treatment near *IL1B* overlap with a candidate cis-regulatory element (cCRE) in A549 cells. B) Changes in expression following HDM treatment and/or introduction of an *IL1B*enhancer plasmid. The shaded region in A) shows a region that was differentially accessible in WT HDM vs. WT saline and in KD HDM vs. WT HDM (decreased in WT HDM in both cases). “A549 cCREs” refer to candidate cis-regulatory elements downloaded from https://screen.encodeproject.org/. Abbreviations: WTHDM = *TET1*intact house dust mite samples, WTSal =*TET1*intact saline samples, KDHDM = *TET1*knockdown house dust mite samples, DARs = differentially accessible regions, C = *TET1* intact saline samples given control plasmid, C+HDM = *TET1* intact house dust mite samples given control plasmid, IL1Benh = *TET1* intact saline samples given *IL1B* enhancer plasmid, IL1Benh+HDM = *TET1* intact house dust mite samples given *IL1B* enhancer plasmid.

HDM and TET1 loss both activate the acute phase response pathway and downregulate the AHR signaling pathway (Supplementary Fig. 4B). *IL1B*, a major stimulator of the acute phase response pathway (69) that has been linked to asthma (70), showed increased gene expression following both TET1 loss and HDM treatment (Supplementary Fig. 4A). Interestingly, a region downstream of *IL1B* became significantly less accessible following HDM treatment and became significantly more accessible in KD HDM compared to WT HDM. This region overlapped with a predicted cCRE from A549 ENCODE data (Fig. 9A). Reporter assays confirm that this region enhances the expression of a florescence gene reporter gene at baseline, to the same extent of the effect of HDM treatment alone (Fig. 9B). These data further support that TET1 may regulate chromatin accessibility and enhancer activity to control gene expression and response to allergen in HBECs.

## Discussion

In this study, we assessed how knockdown of *TET1* and challenge of HBECs with house dust mite extract (HDM, known for its causal role in allergic asthma (62)) affected chromatin accessibility, H3K27ac levels, DNA methylation, and gene expression. One of our most striking findings was how HDM treatment and TET1 status led to highly specific changes in chromatin accessibility. When *TET1* was knocked down or when cells were treated with HDM without knocking down *TET1*, chromatin tended to lose more often than gain accessibility (Fig. 1). When *TET1* was knocked down and cells were treated with HDM, however, chromatin was generally more accessible (Fig. 1). Importantly, a significant portion of chromatin changes resulting from TET1 loss overlapped with chromatin changes following HDM challenges (Fig. 6A). There was also significant overlap between the effects of HDM treatment and *TET1* loss on DNA methylation and H3K27ac levels (Fig. 6B, 6D). There was generally less overlap HDM and TET1 loss-induced alterations to gene expression, primarily due to the relatively small number of DE genes following HDM treatment alone; however, 74 DE genes changed in the same direction in both comparisons (Fig. 6C). Overall, *TET1* knockdown in HBECs had a very similar overall effect to HDM treatment, reinforcing results from our previous studies indicating that TET1 plays a protective role in allergic asthma (9, 68) and supporting a significant mediatory role for TET1 in responses to HDM.

### ET1-mediated changes in chromatin accessibility rarely overlap with TET1-mediated changes in DNA methylation and may represent separate modes of gene regulation.

TET proteins regulate gene expression and cellular function via two main mechanisms: 1) catalytic activity-dependent promotion of DNA demethylation (71) and 2) catalytic activity-independent mechanisms to modify histone marks (72–75). The catalytic activity of TET proteins converts 5mC to 5hmC and eventually results in the removal of DNA methylation (11). It has also been reported that 5mC disrupts CTCF binding (57, 58), while CEBP prefers to bind 5mC compared to 5hmC (76). Therefore, if the enzymatic activity of TET1 plays a major role in HBEC, we would expect to see increased 5mC and decreased 5hmC resulting in increased DNA methylation (77, 78), reduced CTCF binding and increased CEBP binding in *TET1* KD HBECs. Unexpectedly, we observed nearly equal numbers of significantly hypermethylated and hypomethylated regions and no enrichment for either CTCF or CEBP binding sites within DMRs (Supplementary Fig. 6). However, we observed a lack of CTCF and enrichment of CEBP binding sites in regions with changes in chromatin accessibility, which significantly overlapped with regions with H3K27ac changes and had only few overlaps with DMRs. This suggests that the catalytic activity-independent function of TET1 is primarily responsible for the changes in chromatin accessibility and histone marks. One caveat is that WGBS measures the sum of 5mC and 5hmC and cannot distinguish between these two modifications (60). Therefore, our study may have missed regions with simultaneous opposite significant changes (*e.g*. increased 5mC and decreased 5hmC). Indeed, ~ 50% of changes in chromatin accessibly occurred within gene bodies, which are normally highly methylated. Therefore, the two functions of TET1 protein regulation may coordinate to influence the epigenetic landscape and regulate gene expression. Future studies using emerging technologies such as Nanopore sequencing that can separately identify 5mC and 5hmC will be needed to further delineate the relationship between TET1-mediated changes in DNA methylation, histone modifications, TF binding and chromatin accessibility.

### TET1 loss-induced expression changes in aryl hydrocarbon receptor signaling genes may promote allergen-induced inflammation.

We previously showed that TET1 positively regulates the expression of several genes in the aryl hydrocarbon receptor (AhR) signaling pathway at baseline and following HDM challenges in mice (9). This suggests a mechanistic explanation for the exacerbated asthma-like phenotype in TET1-deficient mice following HDM treatment. In our current study in HBECs, DE genes in KD saline vs. WT saline and KD HDM vs. WT HDM were both enriched for the AhR signaling pathway (Supplementary Table 6). Overlapping DE genes for these comparisons included *AHR, ALDH1A1, CYP1B1, GSTO1,* and *IL1B*. The activation z-scores for the AhR signaling pathway were negative for both comparisons, implying that loss of TET1 leads to lower activation of this pathway. The evidence for deactivation, however, was much stronger in the presence of HDM (activation z-scores: −1.81 for KD HDM vs. WT HDM, −0.51 for KD saline vs. WT saline). These *in vitro* HBEC results for genes involved in AhR signaling were generally consistent with the aforementioned *in vivo* mouse study results, further supporting the role of TET1 in regulating this pathway, especially following HDM challenges. Several DE genes in the AhR signaling pathway had changes in accessibility and/or methylation when *TET1* was knocked down (including *AHR, ALDH1A1,* and *NFE2L2* in KD saline vs. WT saline and *ALDH1A1, MGST1,* and *CYP3A5* in KD HDM vs. WT HDM). In addition, the AhR signaling pathway was also enriched in regions with differences in H3K27ac in KD saline vs. WT saline and KD HDM vs. WT HDM (Supplementary Table 11). Assuming a general positive correlation between H3K27ac presence and gene expression, changes in H3K27ac were predicted to reduce the activity of this pathway (activation z-scores: −2.07 for KD saline vs. WT saline, −2.83 for KD HDM vs. WT HDM). Importantly, changes in chromatin accessibility, as well as other epigenetic modifications, following stimulus do not necessarily correlate with immediate changes in gene expression. For example, in other studies, short-term IL-1α treatment led to changes in chromatin accessibility, but the majority of those changes were not correlated with changes in gene expression (58, 79). Nevertheless, although overall gene expression programs differ, the chromatin state in KD saline resembles HDM-treated HBECs (Supplementary Fig. 3), suggesting that a pro-inflammatory chromatin state created by TET1 loss that may promote the response to HDM. In support of this, we only observed a trend of increased expression in *IL33* (a gene known to contribute to airway inflammation following environmental exposures (80)) in WT HDM vs. WT saline, but this gene was significantly upregulated in KD HDM vs. WT HDM.

### Combining TET1 knockdown and HDM treatment led to unique changes in the epigenome, which may influence enhancer activity and contribute to more substantial proinflammatory effects.

*TET1* knockdown and HDM treatment have each individually been linked to asthma (9, 62). These two treatments generally led to similar changes in methylation and accessibility (significantly more overlap than expected by chance, and a high level of concordance in the direction of change). However, in the KD HDM vs. WT HDM comparison, we observed generally increased accessibility in the KD HDM group (Fig. 1). KD HDM samples were closest to WT saline samples in a principal component analysis of the chromatin accessibility data (Supplementary Fig. 3), indicating that combining *TET1* knockdown with HDM treatment reverted many of the changes in accessibility linked to TET1 knockdown alone. While the epigenetic changes following *TET1* knockdown were certainly affected by HDM presence, *TET1* knockdown had a more consistent effect on gene expression. Out of 4457 DE genes in KD HDM vs. WT HDM, 3013 (67.6%) were also DE in KD saline vs. WT saline. Out of these overlapping DE genes, 99% showed consistent changes in direction when *TET1* was knocked down. Therefore, while epigenetic changes (methylation and accessibility) showed vastly different patterns following *TET1* knockdown depending on whether HDM was present, expression changes were very consistent between KD HDM vs. WT HDM and KD saline vs. WT saline.

One chromatin mark that showed general correlation with expression in both KD HDM vs. WT HDM and KD saline vs. WT saline was H3K27ac, a marker for enhancers (63). A previous study found 4321 regions showing differential levels of H3K27ac between asthmatic and healthy bronchial epithelial cells (51), indicating that changes in this chromatin mark could have some link to asthma. Indeed, regions with changing H3K27ac in both KD saline vs. WT saline and KD HDM vs. WT HDM were both enriched for genes in the AhR signaling pathway, a pathway that was inhibited in Tet1 deficient mice when exposed to HDM (9) and known to regulate airway inflammation (81). We also observed changes in H3K27ac peaks at or close to some regions whose chromatin accessibility was regulated by TET1 and HDM in HBECs (Fig. 2B). In addition, computational analysis predicted reduced CTCF binding at these regions. CTCF is a ubiquitously expressed and essential protein that has many functions. CTCF is a well-established regulator of chromatin looping (long distance chromatin interactions) (82–84). Chromatin folding/looping allows regulatory gene elements to act on their targets located long distances away (*in trans*) (85). Allele-specific chromatin remodeling mediated by CTCF was linked to differential expression of genes in the 17q12-q21 locus, which has been linked to childhood-onset asthma (86). In our datasets, we observed significant enrichment for CTCF binding sites, CTCF-bound cCREs, TADs and H3K27ac peaks in A549 cells at regions with TET1- and HDM-mediated chromatin accessible changes (Fig. 7A). Taken together, future studies should assess the effects of other histone modifications, CTCF binding and enhancer function following TET1 loss. Functional studies directly assessing the interplay between TET1, histone modifications, CTCF binding and gene expression could prove fruitful for understanding the role of TET1 in responding to environmental exposures and regulating asthma.

### Study limitations and future directions

This study had some limitations. The sample size for each analysis was relatively small, so we used both stringent quality control and significance cutoffs (*e.g*. we required at least 2-fold change for a significant difference in accessibility). By using these stringent cutoffs, however, we likely missed biologically relevant changes in some genes/regions; however, this approach was deemed necessary to limit false positives. Another limitation was that all HBEC samples were collected at only one time point (after 48 hours of siRNA with 24 hours of treatment with either saline or HDM). It is plausible that for certain genes, 24 hours was enough time for methylation and/or accessibility to change, but not enough time for corresponding gene expression changes. Additionally, there were likely genes that had expression changes at earlier time points that were no longer present at 24 hours, but changes in methylation and/or accessibility persisted. These potential differences in timing might explain, for example, why ~ 74% of differentially expressed genes in KD saline vs. WT saline were not associated with DMRs or differentially accessible regions. Another potential reason for this lack of correlation was our strategy of annotating regions of interest (*e.g*. DMRs or differentially accessible regions) using the closest gene. We likely missed trans-regulatory interactions correlated with gene expression changes because of this. Future studies should include measurements at a variety of time points to enable a more comprehensive understanding of epigenetic regulation of gene expression following *TET1* knockdown and HDM treatment. Another limitation was that whole genome bisulfite sequencing measures the sum of 5mC and 5hmC and cannot differentiate between 5mC and 5hmC (60), so any effect that *TET1* knockdown had on 5mC and 5hmC individual levels was not captured in this study. Lastly, there were several other epigenetic changes that likely affected gene expression. For example, TET1 can directly interact with transcription factors and histone modification enzymes (15), so it would be worthwhile to directly measure how histone modifications and transcription factor binding change following *TET1* knockdown. Collectively, our analyses reveal a new link between TET1, CTCF and enhancers that future studies should expand on to improve our understanding of the role of TET1 in responding to environmental exposures linked to asthma.

### Conclusions

Our genome-scale data analyses indicate that reduction of *TET1*, a key regulator of asthma, in human bronchial epithelial cells leads to global changes in chromatin accessibility, DNA methylation, H3K27ac, and gene expression. These changes were similar to changes observed following house dust mite challenges, a direct cause of allergic asthma development. Altered chromatin accessibility following loss of TET1 was found to potentially affect the binding of CTCF and CEBP. Little overlap was observed between regions of the genome that experienced TET1-induced changes in chromatin accessibility and DNA methylation, supporting a multifaceted gene regulatory role of TET1. Overall, our data shed light on how TET1 regulates critical pathways involved in asthma and response to allergens.

## Data Availability

ATAC-seq, WGBS and RNA-seq data will be deposited to GEO upon manuscript acceptance. The code used for analyses will be uploaded to GitHub.

## Abbreviations

HDM: house dust mite
HBEC: human bronchial epithelial cell
WGBS: whole genome bisulfite sequencing
DMR: differentially methylated region
GSEA: gene set enrichment analysis
cCRE: candidate cis-regulatory element
TF: transcription factor
